# An evaluation of the risk factors associated with implementing projects of health information technology by fuzzy combined ANP-DEMATEL

**DOI:** 10.1371/journal.pone.0279819

**Published:** 2023-02-06

**Authors:** Roxana Sharifian, Farzane Ghasemi, Erfan Kharazmi, Payam Farhadi, Hossein Monem, Nasrin Shokrpour

**Affiliations:** 1 Health Information Management, Faculty of Management and Information Sciences, Health Human Resource Research Center, Shiraz University of Medical Sciences, Shiraz, Iran; 2 Department of Health Information Management and Technology, Medical Informatics, School of Management and Medical Information Sciences, Shiraz University of Medical Sciences, Shiraz, Iran; 3 Department of Health Management, Health Management, Faculty of Management and Medical Information Sciences, Shiraz University of Medical Sciences, Shiraz, Iran; 4 Operational Research, Faculty of Management, Zand Higher Education Institute, Shiraz, Iran; 5 Dept. of Computer Science, Faculty of Paramedical Sciences, Shiraz University of Medical Sciences, Shiraz, Iran; 6 Dept. of English Language, Faculty of Paramedical Sciences, Shiraz University of Medical Sciences, Shiraz, Iran; Sichuan University, CHINA

## Abstract

**Background:**

Application of a Clinical Information System (CIS) like Electronic Patient Record (EPR), PACS system and CPOE has turned into one of the most important criteria of priorities of health care systems. The aims of the clinical information system include improving the physicians’ efficiency level, integrating the caring process, and expanding the fuzzy quality of the services offered to patients. Achievement of these benefits in reality is not an easy task, and there are lots of plans in this field which are doomed to failure. About 50% of the implementation plans of clinical information systems in health care organizations have failed, and this trend is significantly affecting industrial countries. Proper implementation of hospital information systems lies in identifying and assessing the relationships among the most important risk factors of fuzzy. The present study aimed to provide an applicable model for identifying, ranking and evaluating the risk factors associated with projects of clinical information technology in hospitals of Shiraz University of Medical Sciences.

**Method:**

This is an applied study which evaluates the risk factors associated with implementation of clinical information technology projects in hospitals of Shiraz Medical Sciences University. The participants consisted of professionals and senior experts of clinical information technology. Fuzzy logic was used in this study. We also applied ANP-DEMATEL combined model with fuzzy procedure to provide the analytic model of the study

**Results:**

According to the study findings, lack of top-executive supports, and unstable organizational environment were the two most important risk factors, while the main organizational factors and technology were also highly important. In addition, the factors associated with technology had the highest influence on the other studied risk factors.

**Conclusion:**

Hospital authorities can benefit from this proposed model to reduce the risk of implementing the projects of clinical information technology and improve the success coefficient of the risk of such projects.

## Background

Application of a Clinical Information System (CIS) like Electronic Patient Record (EPR), PACS system, and CPOE has turned into one of the most important criteria of the priorities of healthcare systems. The aims of the clinical information system include improving the physicians’ efficiency level, integrating the caring process, reducing the clinical errors, increasing the patients’ access to health services, practicing remote care and continuity of services, reducing healthcare costs, and expanding the quality of services offered to patients. Reaching these benefits in reality is not an easy task, and there are a great number of plans in this field which are doomed to failure [1–4]. For example, 50% of the implementation plans of clinical information systems in health care organizations have been reported to have failed [5, 6]. It seems that this trend is significantly affecting the industrial countries [7]. In addition, scientific studies reveal that many ICIS projects have failed due to problems like insufficient resources, conflicts in organizational mission, need to a comprehensive database, issues associated with data ownership, finical problems, organizational and professional resistance, project management, and high expenses of information technology [6, 8–11]. Brender et al. performed a Delphi study with the aim of recognizing the success and failure criteria for CIS projects. They conducted four studies on 19 subjects at the European Federation for Medical Informatics (EFMI) and totally 27 failure criteria by the panel of academics. Among them, the most significant factors were poor response time, misalignment of the system and the organization, and project structure, and/or work procedures [12]. Chiang and Starren suggested a conceptual framework for understanding the risk factors related to small Electronic Medical Record (EMR) projects. According to their assessment of a delayed EMR project in a small specialty practice, these scholars found that the results were mainly determined by the lack of a project champion, changes to membership on the project teams, and failure to understand the project requirements [13]. Although recent improvements in information technology, especially network technology and clinical information standards, have eliminated some of these barriers, expansion of such systems is yet very complicated and their implementation is highly risky (1). There are many risk factors including human, organization, and technological factors which influence the hospital information technology systems. Pare et al. conducted a Delphi research on 21 participants selected among the experts of clinical information system projects in Canada to identify the risk factors of clinical information systems and measure the relative importance of each risk factor. They recognized 23 risk factors and introduced lack of commitment from upper management, and lack of a project champion as the two most important risk factors [14]. However, in evaluating the hospital information system only one or two factors are considered, while evolution of hospital information systems is a multifactor analysis [15, 16]. Yucel et al. performed a study to provide a model of evolution risk of implementing information systems in a hospital in Turkey. They found that the most important factors in implementing hospital information system were technology factors, usability, adjustment, user participation, and ease of use [17]. Sicotte et al. designed a risk framework to examine the obstacles of successfully implementing inter-organizational CIS. To test the applicability of the model, they conducted a longitudinal multiple-case study of two large-scale projects and assessed the variations in risk factor measures throughout both projects to determine their influence on the project’s success. Their analysis showed that the proposed framework was very robust and suitable for conducting a thorough risk analysis of this particular type of system [1]. Therefore, identifying the most important risk factors of implementations of clinical information technology systems seems necessary. Therefore, the current study was performed with the aim of ranking the most important risk factors associated with implementation of clinical information systems via a combined procedure of DEMATEL and analysis network process.

Successful implementation of clinical information technology has always been difficult. There are challenges and barriers to effective interoperability [18]. Association of medical informatics with a comprehensive view of software engineering and failure of software projects can be beneficial in investigating the reasons and potential solution. Although this is an important issue, there are few studies on providing a model, integrating the risk factors in projects of clinical information technology, and creating a managerial dashboard for project managers. The authors have investigated previous research on implementation of information technology systems, clinical information systems, as well as health informatics. The rationale for the use of fuzzy combined ANP-DEMATEL in this study was classification and localization of the risks extracted from previous studies. Also, fuzzy DEMANTEL technique is efficient to be used for detection of causal relationship between the risks; for the same purpose, it was used in this study. Fuzzy ANP technique is a useful instrument for determining the weight of the risks in complex conditions. Therefore, we used it in the study. The most important identified risk factors or the factors affecting successful implementation of clinical information technology are shown in Table 1.

**Table 1:** Risk dimensions, risk factors, and empirical support.

Risk Dimensions	Risk Factors	Empirical Support
Technological(C1)	• Lack of a secure and reliable network (S11)	[1, 14]
	• Introduction of new technology (S12)	[14, 19–24]
	• Use of diverse and incompatible hardware and Software (need for system integration) (S13)	[1, 25]
Human (C2)	• Resistance to change in general (S21)	[1, 7, 13, 19–21, 23]
	• Lack of computer skills and knowledge (S22)	[1, 7, 19, 22, 25–27]
	• Users with negative attitudes toward the project (S23)	[1, 20]
Usability (C3)	• Lack of perceived system ease of use (S31)	[1, 7, 17]
	• Lack of perceived system usefulness (S32)	
Managerial & project team (C4)	• Inadequate project team members (S41)	[19, 20, 27, 28]
	• Lack of top-executive support (S42)	[14, 21–23, 26, 28, 29]
	• Lack of required knowledge/skills in the project personnel (S43)	[19, 20, 23]
Organizational (C5)	•Lack of local personnel knowledgeable in IT(S51)	[1, 7, 19, 22]
	•Organizational instability (S52)	[7, 19, 20]
	•Change in organizational management during the project (S53)	[19–21]
Strategic & political (C6)	• Inter organizational conflicts (S61)	[1, 22, 26]

## Methods

This is an applied study which evaluates the risk factors associated with implementation of clinical information technology projects in hospitals of Shiraz Medical Sciences University. The study population consisted of all professionals and senior experts of clinical information technology. The participants gave their written informed consent before they took part in the study.

Fuzzy logic was used since the traditional process of quantification of the peoples’ views does not allow reflection of human thinking style, and this approach is more adjusted with verbal and sometimes vague human statements [30]. The S1 Fig displays the variables chosen for the analysis.

**Fig 1:**
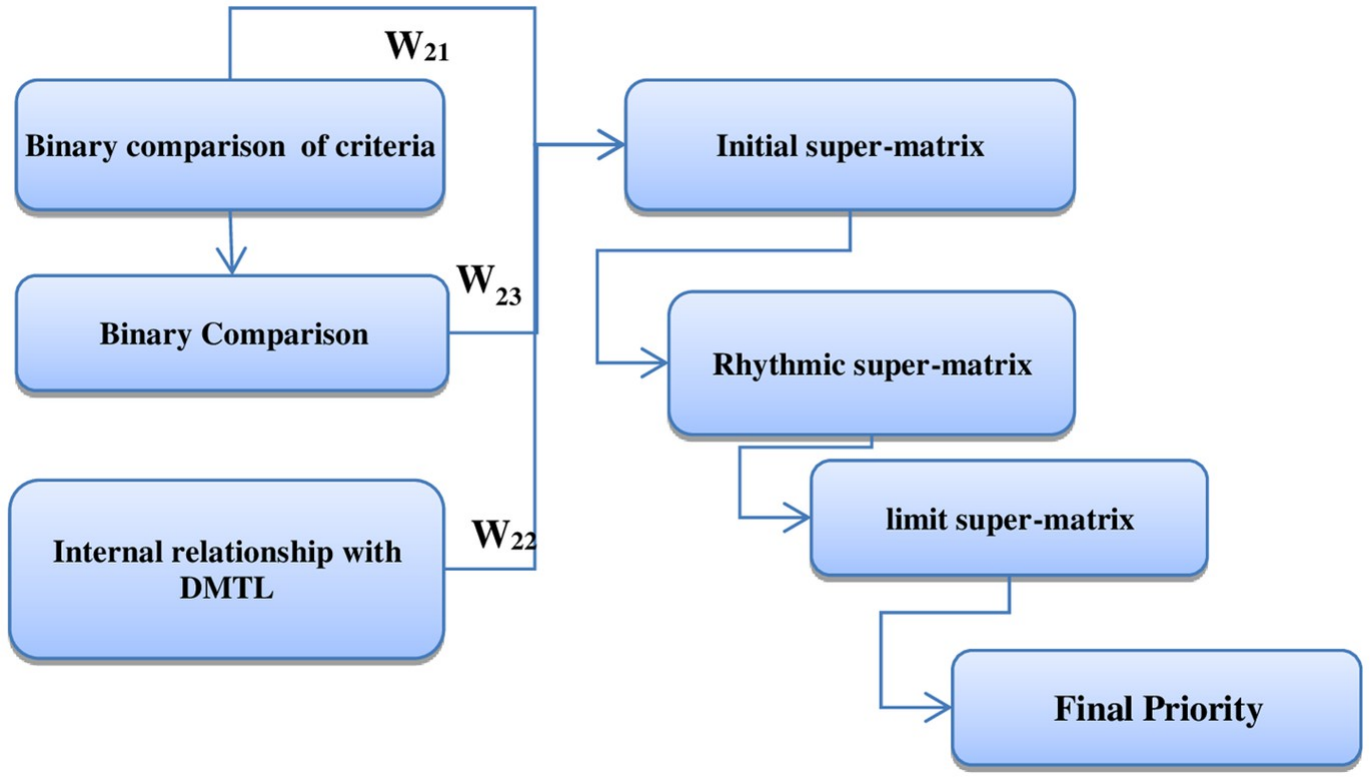
Proposed framework of the ANP-DEMATEL combined approach.

Combined approach of ANP-DEMATEL has been used to rank various risk factors associated with clinical information technology (Fig 1). This combined approach has formerly been used in other studies [31–34].

This study has applied fuzzy DEMATEL approach to identify the pattern of casual relationships among the main criteria. The output of DEMATEL technique has been used as the matrix of internal relationships among the main criteria (*W*_22_).

Fuzzy DEMATEL technique

Implementation steps of fuzzy DEMATEL technique are as follows:

Step 1: Calculation of direct access matrix (***X***)
  First, the experts’ views were fuzzied with the fuzzy continuum in Table 2.
Step 2: Normalizing the matrix
Step 3: Computing the full contact matrix

**Table 2:** Fuzzy continuum and DEMATEL technique, source [35].

Importance	Definition
(0.0, 0.1, 0.3)	No effect
(0.1, 0.3, 0.5)	Little effect
(0.3, 0.5, 0.7)	Medium effect
(0.5, 0.7, 0.9)	Strong effect
(0.7, 0.9, 1.0)	Very strong effect

After computing the full contact matrix, dis-fuzzy becomes possible. The new matrix is the same full contact cut matrix and can be used to calculate the casual relationships. There are various methods for dis-fuzzy including surface center method [36].

### Process of fuzzy network analysis

Network analysis approach was introduced by Saaty in 1986. ANP is the extended form of AHP. In many cases, hierarchical relationships among the criteria are not necessarily dominated, and there are internal relationships among and inside the clusters; therefore, this technique is called analysis network process [37]. Saaty’s 9-degree continuum was used for paired comparison of the main criteria and elements of each of these main criteria. In addition, Saati’s 9-degree continuum was applied for fuzzing verbal statements, as shown in Table 3.

**Table 3:** Fuzzy continuum equal to Saaty’s 9-degree scale.

Reverse fuzzy	importance	Explanation
(1,1,1)	(1, 1, 1)	Preferred Equally
$\left(\frac{1}{3},\frac{1}{2},1\right)$	(1, 2, 3)	Average effect
$\left(\frac{1}{4},\frac{1}{3},\frac{1}{2}\right)$	(2, 3, 4)	Preferred moderately
$\left(\frac{1}{5},\frac{1}{4},\frac{1}{3}\right)$	(3, 4, 5)	Average effect
$\left(\frac{1}{6},\frac{1}{5},\frac{1}{4}\right)$	(4, 5, 6)	Preferred Strongly
$\left(\frac{1}{7},\frac{1}{6},\frac{1}{5}\right)$	(5, 6, 7)	Average effect
$\left(\frac{1}{8},\frac{1}{7},\frac{1}{6}\right)$	(6, 7, 8)	very strongly Preferred
$\left(\frac{1}{9},\frac{1}{8},\frac{1}{7}\right)$	(7, 8, 9)	Average effect
$\left(\frac{1}{9},\frac{1}{9},\frac{1}{9}\right)$	(9, 9, 9)	Extremely Preferred

The matrix of fuzzy paired comparison of the $\left[\tilde{A}\right]$ element is shown in Table 4.

**Table 4:** The matrix of fuzzy paired comparison of the $\left[\tilde{A}\right]$ element.

	C1	…	C_n_	Fuzzy expansion of each row
C1	(1,1,1)			${\tilde{S}}_{1}=\sum_{j=1}^{n}{\tilde{a}}_{1j}$
⋮	⋮	⋮	⋮	⋮
C_n_			(1,1,1)	${\tilde{S}}_{n}=\sum_{j=1}^{n}{\tilde{a}}_{nj}$
				$\left[\sum_{i=1}^{n}{\tilde{S}}_{i}\right]$

In the next phase, inharmonic super-matrix turns into a harmonic (normal) one by applying the concept of normalization. In the harmonic super-matrix, the sum of elements of all columns becomes 1. The next step is to calculate the limit matrix. This super-matrix is obtained by the power of all elements of the harmonic super-matrix. This act is repeated until all the elements of the super-matrix become similar. In this state, all the elements of the super-matrix will be zero and the only elements of sub-criteria become a number that is repeated in all rows of that element. Therefore, the final weight of the criteria is determined.

## Result

**Fig 2:**
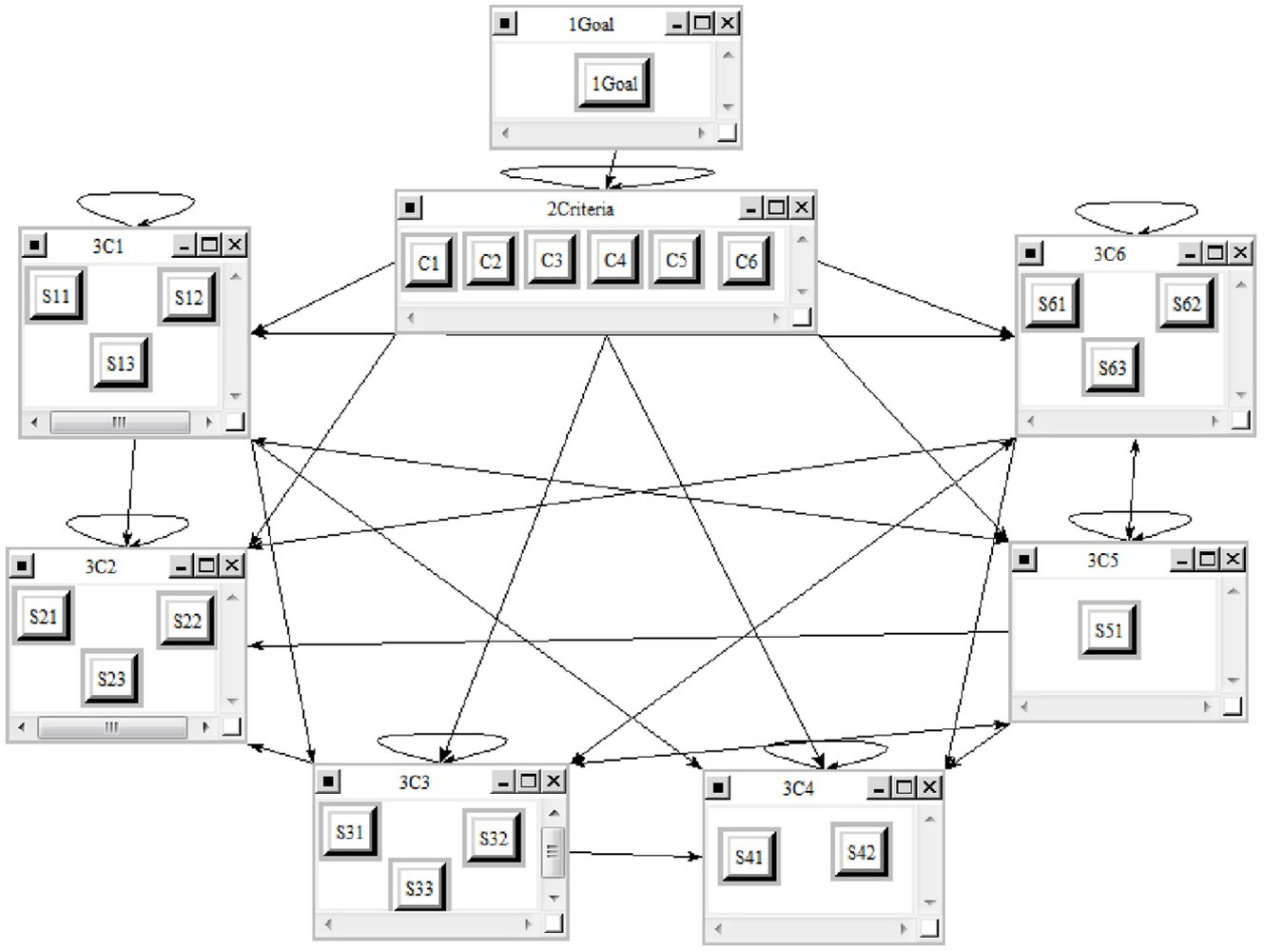
ANP graph, priority of the indexes and options in Super-Decision software identifying internal relationships of the main criteria.

To apply the ANP-DEAMTEL combined approach, in a case study, we evaluated the risks of implementing the projects of clinical information technology in hospitals of Shiraz Medical Sciences University. The network pattern of this model was designed using ANP technique on Super-Decision software. Based on this model, the graph of analysis network process (ANP) is shown in Fig 2.

Based on the proposed model in this study, the first phase identified the internal relationships of the main criteria. Experts’ views were fuzzied by the fuzzy continuum of Table 1. By using relationship 2, the matrix of fuzzy direct relation $\tilde{X}$ was formed (Table 5).

**Table 5:** Computing the matrix of fuzzy direct relationship.

$\tilde{X}$	C1	C2	C3	C4	C5	C6
C1	(0, 0.1, 0.3)	(0.36, 0.56, 0.76)	(0.62, 0.82, 0.95)	(0.44, 0.64, 0.83)	(0.15, 0.34, 0.53)	(0.26, 0.46, 0.65)
C2	(0.25, 0.44, 0.64)	(0, 0.1, 0.3)	(0.5, 0.7, 0.86)	(0.3, 0.5, 0.7)	(0.03, 0.16, 0.36)	(0.24, 0.44, 0.64)
C3	(0.08, 0.22, 0.42)	(0.16, 0.36, 0.56)	(0, 0.1, 0.3)	(0.22, 0.42, 0.62)	(0.5, 0.7, 0.89)	(0.56, 0.76, 0.89)
C4	(0.11, 0.3, 0.5)	(0.42, 0.62, 0.82)	(0.5, 0.7, 0.89)	(0, 0.1, 0.3)	(0.22, 0.42, 0.62)	(0.44, 0.64, 0.83)
C5	(0.01, 0.12, 0.32)	(0.25, 0.44, 0.64)	(0.56, 0.76, 0.92)	(0.45, 0.64, 0.8)	(0, 0.1, 0.3)	(0.5, 0.7, 0.86)
C6	(0.11, 0.3, 0.5)	(0.36, 0.56, 0.76)	(0.5, 0.7, 0.86)	(0.32, 0.52, 0.71)	(0.5, 0.7, 0.89)	(0, 0.1, 0.3)

For normalizing the values, ${\tilde{a}}_{i}^{\left(k\right)}$ and ${\tilde{b}}^{\left(k\right)}$ are computed. By dividing the elements of matrix $\tilde{X}$ on the utmost values of Σ*u*_*ij*_, $\tilde{N}$ fuzzy normal matrix was obtained, as shown in Table 6:

$${\tilde{b}}^{\left(k\right)}=4.02;\tilde{N}=\frac{1}{4.02}*\tilde{X}$$

**Table 6:** Computation of the normal matrix of fuzzy direct contact.

$\tilde{N}$	C1	C2	C3	C4	C5	C6
C1	(0, 0.02, 0.07)	(0.09, 0.14, 0.19)	(0.15, 0.2, 0.24)	(0.11, 0.16, 0.21)	(0.04, 0.08, 0.13)	(0.06, 0.11, 0.16)
C2	(0.06, 0.11, 0.16)	(0, 0.02, 0.07)	(0.12, 0.17, 0.21)	(0.07, 0.12, 0.17)	(0.01, 0.04, 0.09)	(0.06, 0.11, 0.16)
C3	(0.02, 0.05, 0.1)	(0.04, 0.09, 0.14)	(0, 0.02, 0.07)	(0.05, 0.1, 0.15)	(0.12, 0.17, 0.22)	(0.14, 0.19, 0.22)
C4	(0.03, 0.07, 0.12)	(0.1, 0.15, 0.2)	(0.12, 0.17, 0.22)	(0, 0.02, 0.07)	(0.05, 0.1, 0.15)	(0.11, 0.16, 0.21)
C5	(0, 0.03, 0.08)	(0.06, 0.11, 0.16)	(0.14, 0.19, 0.23)	(0.11, 0.16, 0.2)	(0, 0.02, 0.07)	(0.12, 0.17, 0.21)
C6	(0.03, 0.07, 0.12)	(0.09, 0.14, 0.19)	(0.12, 0.17, 0.21)	(0.08, 0.13, 0.18)	(0.12, 0.17, 0.22)	(0, 0.02, 0.07)

To calculate the full contact matrix, we used N × (*I* − *N*)^−1^ relationship, as shown in Table 7.

**Table 7:** Full contact matrix of the main criteria.

$\tilde{T}$	C1	C2	C3	C4	C5	C6
C1	(0.021, 0.167, 2.407)	(0.134, 0.371, 3.438)	(0.223, 0.516, 4.192)	(0.155, 0.399, 3.531)	(0.092, 0.323, 3.305)	(0.133, 0.401, 3.742)
C2	(0.074, 0.22, 2.21)	(0.038, 0.223, 2.949)	(0.175, 0.43, 3.703)	(0.11, 0.323, 3.108)	(0.052, 0.242, 2.894)	(0.11, 0.342, 3.314)
C3	(0.035, 0.178, 2.261)	(0.084, 0.304, 3.155)	(0.075, 0.326, 3.758)	(0.102, 0.329, 3.242)	(0.165, 0.377, 3.15)	(0.189, 0.433, 3.528)
C4	(0.046, 0.208, 2.419)	(0.143, 0.372, 3.4)	(0.191, 0.476, 4.119)	(0.051, 0.268, 3.362)	(0.105, 0.33, 3.277)	(0.166, 0.424, 3.724)
C5	(0.023, 0.166, 2.319)	(0.109, 0.334, 3.28)	(0.207, 0.486, 4.024)	(0.155, 0.386, 3.388)	(0.059, 0.26, 3.127)	(0.186, 0.439, 3.642)
C6	(0.045, 0.21, 2.448)	(0.132, 0.366, 3.432)	(0.196, 0.488, 4.169)	(0.13, 0.373, 3.502)	(0.168, 0.395, 3.376)	(0.073, 0.317, 3.658)

Surface-center approach was applied to dis-fuzzing. Results of cutting the full contact matrix are summed up in Table 8.

**Table 8:** The pattern of affecting and being affected of the main criteria.

D-R	D+R	R	D	Criteria	
2.70	13.00	5.15	7.85	C1	Technological
-0.58	14.26	7.42	6.84	C2	Human
-2.02	16.48	9.25	7.23	C3	Managerial & project team
0.06	15.33	7.64	7.69	C4	Usability
0.30	14.76	7.23	7.53	C5	Strategic & political
-0.45	16.10	8.27	7.83	C6	Organizational

In the full contact matrix, the sum of elements in each row (D) shows the affecting power of that factor on other factors in the system, and the sum of elements in each column (R) indicates how much that element is affected. Moreover, the column (D+R) displays the power of affecting and being affected of an element in the system, while the column (D-R) is the indictor of the affecting power of each element. Generally, if D-R is positive, the variable is casual; otherwise, it is effect.

Internal relationships of sub-criteria were investigated by the same method. Due to the prolixity and resemblance of the phases, only the dis-fuzzed full contact matrix, as W_33_, was used (Table 9).

**Table 9:** Internal relationships of the sub-criteria.

Sub-criteria	Symbol	D	R	D+R	D-R	Rank
Lack of a secure and reliable network	S11	2.90	2.44	5.35	0.46	5
Use of diverse and incompatible hardware and software	S12	3.16	2.32	5.48	0.84	4
Introduction of new technology	S13	2.98	2.95	5.93	0.04	7
Resistance to change in general	S21	2.80	3.98	6.78	-1.17	15
Lack of computer skills and knowledge	S22	2.95	2.90	5.85	0.05	6
Users with negative attitudes toward the project	S23	2.89	4.02	6.91	-1.12	14
Lack of top-executive support	S31	3.11	3.78	6.89	-0.68	13
Lack of required knowledge/skills in the project personnel	S32	3.44	2.07	5.51	1.37	1
Inadequate project team members’ skills and knowledge	S33	3.13	2.21	5.34	0.92	2
Lack of perceived system ease of use	S41	3.12	3.23	6.34	-0.11	8
Lack of perceived system usefulness	S42	3.01	3.33	6.35	-0.32	10
Inter organizational conflicts	S51	2.87	3.33	6.20	-0.45	12
Organizational instability	S61	2.99	3.36	6.35	-0.37	11
Change in organizational management during the project	S62	2.61	2.90	5.51	-0.29	9
Lack of local personnel knowledgeable in IT	S63	3.43	2.58	6.01	0.85	3

### Paired comparison of elements

In the next phase, we compared the main elements based on the goal in a paired manner. Since there were 6 criteria, 10 paired comparisons were performed on the experts’ views. Then, these views were fuzzied and summed with the geometric mean. The matrix of the paired comparison is illustrated in Table 10.

**Table 10:** The matrix of the paired comparison of the main criteria.

	C1	C2	C3	C4	C5	C6
C1	(1, 1, 1)	(2.35, 1.94, 1.52)	(0.82, 0.7, 0.6)	(1.15, 0.92, 0.73)	(2.26, 1.89, 1.58)	(0.51, 0.43, 0.36)
C2	(0.66, 0.52, 0.43)	(1, 1, 1)	(0.39, 0.33, 0.29)	(1.62, 1.38, 1.17)	(0.97, 0.8, 0.68)	(0.55, 0.49, 0.44)
C3	(1.66, 1.43, 1.22)	(3.42, 3, 2.59)	(1, 1, 1)	(2.78, 2.36, 1.89)	(2.7, 2.25, 1.79)	(0.98, 0.79, 0.61)
C4	(1.37, 1.09, 0.87)	(0.86, 0.72, 0.62)	(0.53, 0.42, 0.36)	(1, 1, 1)	(1.08, 0.88, 0.73)	(0.48, 0.38, 0.31)
C5	(0.63, 0.53, 0.44)	(1.47, 1.25, 1.03)	(0.56, 0.44, 0.37)	(1.37, 1.13, 0.92)	(1, 1, 1)	(0.48, 0.39, 0.32)
C6	(2.78, 2.35, 1.94)	(2.26, 2.04, 1.81)	(1.64, 1.27, 1.02)	(3.19, 2.62, 2.08)	(3.1, 2.58, 2.08)	(1, 1, 1)

Therefore, the fuzzy expansion of each row elements is as follows:

$$\sum_{j=1}^{6}{\tilde{x}}_{1j}=(5.78,6.87,8.1)$$

$$\sum_{j=1}^{6}{\tilde{x}}_{2j}=(4.01,4.52,5.18)$$

$$\sum_{j=1}^{6}{\tilde{x}}_{3j}=(9.1,10.82,12.55)$$

$$\sum_{j=1}^{6}{\tilde{x}}_{4j}=(3.89,4.5,5.32)$$

$$\sum_{j=1}^{6}{\tilde{x}}_{5j}=(4.09,4.74,5.52)$$

$$\sum_{j=1}^{6}{\tilde{x}}_{6j}=(9.94,11.86,13.97)$$

The sum of elements in the preference column of the main criteria is:

$$\sum_{i=1}^{6}\sum_{j=1}^{6}{\tilde{x}}_{ij}=(36.81,43.31,50.64)$$

To normalize the preferences of each criterion, the reverse of the sum should be calculated:

$${\left(\sum_{i=1}^{n}\sum_{j=1}^{n}{\tilde{x}}_{ij}\right)}^{-1}=\left(0.02,0.023,0.027\right)$$

$${S_{k}=\sum_{i=1}^{n}M*(\sum_{i=1}^{n}\sum_{j=1}^{n}{\tilde{x}}_{ij})}^{-1}$$

Results of normalization of the obtained values are as follows:

$$\begin{array}{l} \text{C}1=\left(0.12,0.16,0.22\right) \end{array}$$

$$\begin{array}{l} \text{C}2=\left(0.08,0.1,0.14\right) \end{array}$$

$$\begin{array}{l} \text{C}3=\left(0.18,0.25,0.34\right) \end{array}$$

$$\begin{array}{l} \text{C}4=\left(0.08,0.1,0.14\right) \end{array}$$

$$\begin{array}{l} \text{C}5=\left(0.08,0.11,0.15\right) \end{array}$$

$$\begin{array}{l} \text{C}6=\left(0.2,0.27,0.38\right) \end{array}$$

Each of the obtained values is the fuzzied and normalized weight associated with the main criteria.

### Final priority of the indexes by FDANP technique

In order to determine the final weight, we entered the comparison output of the main criteria based on the goal and internal relationships among the criteria into the initial or inharmonic super-matrix. This super-matrix was first normalized and turned into a harmonic one. Then, the final weights of each index were determined via computing the limit form of the super-matrix (Table 11).

**Table 11:** The final weight of the indices based on the limit super-matrix.

Final criteria	Symbol	Total weight	Normal weight	Rank
Lack of a secure and reliable network	S11	0.0219	0.04	9
Use of diverse and incompatible hardware and software	S12	0.0092	0.02	14
Introduction of new technology	S13	0.0043	0.01	15
Resistance to change in general	S21	0.0168	0.03	11
Lack of computer skills and knowledge	S22	0.0116	0.02	13
Users with negative attitudes toward the project	S23	0.0235	0.05	8
Lack of top-executive support	S31	0.0857	0.17	1
Lack of required knowledge/skills in the project personnel	S32	0.0678	0.14	3
Inadequate project team members’ skills and knowledge	S33	0.0165	0.03	12
Lack of perceived system ease of use	S41	0.0194	0.04	10
Lack of perceived system usefulness	S42	0.0249	0.05	7
Inter organizational conflicts	S51	0.0283	0.06	6
Organizational instability	S61	0.0741	0.15	2
Change in organizational management during the project	S62	0.0595	0.12	4
Lack of local personnel knowledgeable in IT	S63	0.0364	0.07	5

## Discussion

In recent years, researchers have used multifactorial decision-making techniques like DEMATEL to find out important variables in health projects [38–40]. According to the results of the DEMATEL technique, the factor “lack of top-executive supports” had the most affecting and affected power, while the factor “lack of required knowledge/skills in the project personnel” was the most important causal factor.

In a study conducted by Shah et al. on electronic health records, the role of human forces, especially top management, in HIT projects has been emphasized [41]. In addition, a look at the outcomes of the analysis network process revealed that the three factors of “lack of top-executive supports”, “unstable organizational environment’, and “lack of professional skills of the project team” had the most importance, respectively, and should be specifically considered in implementation of clinical information technology projects. Edsar et al. considered the top management team, information technology department, and organization at large as significant factors in this regard [42].

The findings of the current study can be used as a managerial dashboard of the risk factors associated with implementation of clinical information technology. It should also be noted that considering the internal relationships among the variables makes the importance and ranking of the indexes more trustable. Applying the suggested model helps the managers to reduce the risk coefficient associated with implementation of clinical information technology projects.

Another finding of the present study was that technology had the strongest affecting power, showing the highest degree of being affected for the project management. In other words, technology, usability, and strategic-political factors are casual variables, while the users, organizations, and project management are the cause. Many studies have been carried out in the world on the use of HIT in health sectors [43–45], and in most of them the importance of IT in health systems have been highlighted.

## Conclusion

Now, identifying the risk factors associated with implementation of clinical information technology in health care organization has turned into a major challenge for managers, physicians, and experts of information technology. The risks differ greatly based on their severity levels and outcomes; therefore, identifying and managing high level risks are very important. The authors believe that by having a comprehensive list of risk factors and their determined importance level, those managers and physicians who participate in implementation of clinical information systems may monitor these factors and their consequences more efficiently. The main criteria of the identified risks in this study include technology, human, usability, managerial and project team, organizational and strategic-political aspects which totally have 15 sub-criteria mentioned in many studies on information technology and medical informatics.

## Supporting information

S1 Fig The variables used for analysis .
(DOCX)

S1 Data (XLSX)

S2 Data (XLSX)

S3 Data (XLSX)
