# Supplementary material for: An evaluation of the risk factors associated with implementing projects of health information technology by fuzzy combined ANP-DEMATEL
Source: PLoS One. 2023 Feb 6;18(2):e0279819. doi: 10.1371/journal.pone.0279819 (PMC9901768; doi:10.1371/journal.pone.0279819)
Supplement: S1 Fig — . (DOCX) [file pone.0279819.s001.docx]

Supporting Flowchart 1: The variables used for analysis

phase1 11 1

Phase2

Phase3

Phase4

Extraction of risks

Summarization & localization

Determination of causal relation

Prioritization & weight allocation

Previous studies

DELPHI FUZZY

FUZZY DEMATEL

FUZZY ANP
